# Current News

**Published:** 1902-04

**Authors:** 


### Current News.

### MISSISSIPPI BOARD OF DENTAL EXAMINERS.

THE Mississippi Board of Dental Examiners will hold its annual session in the city of Jackson, on the 6th of May, 1902.

W. R. WRIGHT,
*Secretary.*

JACKSON, MISS.

### NEW JERSEY STATE BOARD OF REGISTRATION AND EXAMINATION IN DENTISTRY.

The New Jersey State Board of Registration and Examination in Dentistry will hold their next examination on the following dates: Monday, July 7, Tuesday, 8, Wednesday, 9, 1902, at the office of the secretary, J. Allen Osmun, 588 Broad Street, Newark, N. J. All applicants for examination must have their application in two weeks prior to the examination.

J. Allen Osmun,
*Secretary.*

---

### CONNECTICUT STATE DENTAL ASSOCIATION.

The thirty-eighth annual meeting of the Connecticut State Dental Association will be held at Hartford, Tuesday and Wednesday, May 20 and 21, 1902. Every effort is being made to have a large and interesting meeting. At last year's convention over two hundred were present. A larger attendance is expected this year.

Exhibitors desiring space will communicate with the chairman of the Executive Committee, George O. McLean, Hartford, Conn.

Frederick Hindsley,
*Secretary.*

Bridgeport, Conn.

---

### BOARD OF DENTAL EXAMINERS OF PENNSYLVANIA.

The Board of Dental Examiners of Pennsylvania will conduct examinations simultaneously in Philadelphia and Pittsburg, May 6 and 9, 1902.

For papers or further information, apply to Hon. James W. Latta, Secretary Dental Council, Harrisburg, Pa.

G. W. Klump,
*Secretary.*

Williamsport, Pa.

### SEVENTH DISTRICT DENTAL SOCIETY OF THE STATE OF NEW YORK.

The thirty-fourth annual meeting of the Seventh District Dental Society of the State of New York will be held at the Osborn House, Rochester, New York, April 8 and 9, 1902.

A number of valuable papers will be read and a great many clinics given. All members of the profession are cordially invited.

F. Messerschmitt,
*Secretary.*

138 Main Street, East, Rochester, N Y.

---

### ALUMNI ASSOCIATION, NEW YORK COLLEGE OF DENTISTRY.

At the annual meeting of the Alumni Association of the New York College of Dentistry, held on January 15, 1902, at the Hotel Majestic, the following, all of New York City, were elected to serve during the ensuing year:

President, Dr. John I. Hart; First Vice-President, Dr. Edward Fox; Second Vice-President, Dr. H. R. Armstrong; Secretary, Dr. J. Ostram Taylor; Treasurer, Dr. F. A. Chicherio; Curator, Dr. F. J. McLaren.

*Executive Committee.*—Dr. W. C. Deane, Chairman; Dr. Finn Fossume, Dr. B. C. Nash.

J. Ostram Taylor,
*Secretary.*

---

### READING DENTAL SOCIETY.

At the January meeting of the Reading Dental Society the following officers were elected for 1902:

President, Dr. E. W. Bohn; Vice-President, Dr. George S. Schlegel; Treasurer, Dr. Elwood Tate; Secretary, Dr. C. R. Scholl.

*Executive Committee.*—Dr. S. E. Tate, Dr. W. D. De Tony, Dr. H. L. Cleaver.

The fourth annual banquet took place on February 6. Professor T. C. Stellwagen, guest of honor, was the essayist.

C. R. SCHOLL,
*Secretary.*

---

### HARVARD ODONTOLOGICAL SOCIETY.

THE twenty-fourth annual meeting of the Harvard Odontological Society was observed as "Ladies' Night" at Young's Hotel, Boston, Mass., on Saturday evening, March 1, 1902.

After the dinner there was an oration by Herbert A. Reed, D.M.D., entitled "Some Cheerful Facts about our Great Country," followed by an illustrated lecture by Rev. Peter MacQueen, M.A., of Boston, entitled "Beautiful Russia, the Home of the White Czar."

Music was furnished by Mrs. Jeanette Bradbury Chase and Miss Clara E. Shedd.

The officers elected for the ensuing year are, President, Julius G. W. Werner, D.M.D.; Recording Secretary, John W. Estabrooks, D.M.D.; Corresponding Secretary, Arthur H. Stoddard, D.M.D.; Treasurer, Allen S. Burnham, D.M.D.; Editor, Harry W. Haley, D.M.D.

*Executive Committee.*—John W. Estabrooks, D.M.D., Chairman; William P. Cooke, D.M.D., Lyman F. Bigelow, D.M.D.

JOHN W. ESTABROOKS,
*Secretary.*

---

### INSTITUTE OF DENTAL PEDAGOGICS.

AT the close of the ninth annual meeting of the Institute of Dental Pedagogics, held at Pittsburg, Pa., December 31, 1901, and January 1 and 2, 1902, the following officers were elected for the ensuing year:

President, Hart J. Goslee, Chicago, Ill.; Vice-President, J. D.

Patterson, Kansas City, Mo.; Secretary and Treasurer, H. B. Tileston, Louisville, Ky.; Master of Exhibits, D. M. Cattell, Chicago, Ill.

*Executive Board.*—W. Earl Willmott, Toronto, Canada; W. H. Whitslar, Cleveland, Ohio; D. R. Stubblefield, Nashville, Tenn.

D. R. Stubblefield was elected on the Executive Board for the term of three years, to succeed D. M. Cattell, whose term expired.

It was decided to hold the next meeting in Chicago, during the holidays.

H. B. Tileston,
*Secretary and Treasurer.*

314 Equitable Building, Louisville, Ky.

---

### CENTRAL DENTAL ASSOCIATION OF NORTHERN NEW JERSEY.

At the annual meeting of the Central Dental Association of Northern New Jersey, held on Saturday evening, February 15, 1902, the election of officers resulted as follows:

President, J. W. Fisher, East Orange; Vice-President, William H. Pruden, Paterson; Secretary, Frederick W. Stevens, Newark; Treasurer, Charles A. Meeker, Newark.

*Executive Committee.*—C. F. Alfred Hane, Jersey City; C. W. Hoblitzell, Jersey City; F. Edsall Riley, Newark; W. Moore Gould, Newark; H. P. Marshall, Newark.

Frederick W. Stevens,
*Secretary.*

588 Broad Street, Newark, N. J.

---

### IOWA STATE DENTAL SOCIETY.

The annual meeting of the Iowa State Dental Society will be held in Des Moines, May 6, 7, 8, and 9, 1902. All reputable members of the profession are invited to be present.

I. C. Brownlie,
*Secretary.*

### TEXAS STATE DENTAL ASSOCIATION.

The annual meeting of the Texas State Dental Association will be held in the city of Waco, May 13, 14, and 15, 1902. The Executive Committee promise a fine programme, and the meeting is expected to be the largest in the history of the Association.

BUSH JONES,
*Secretary.*

---

### MISSOURI STATE DENTAL ASSOCIATION.

The thirty-eighth annual meeting of the Missouri State Dental Association will be held at Jefferson City, Mo., May 21, 22, and 23, 1902. The business meetings of the Association will be held in the Legislative Hall of the State Capitol. The clinics being held in the State Penitentiary insures an abundance of clinical material. The papers to be read before the Association are of a most interesting character. The meeting bids fair to be one of the best ever held in the State. It is certainly to be hoped that with a change in the time of holding the meeting, and many other attractive features, the attendance should be all that could be desired.

J. H. KENNERLY,
*Corresponding Secretary.*

---

### AMERICAN SOCIETY OF ORTHODONTISTS.

The second annual meeting of the American Society of Orthodontists will be held in Philadelphia, October 8, 9, and 10, 1902. Complete announcement in due time.

MILTON T. WATSON,
*Secretary.*

270 WOODWARD AVENUE, DETROIT.

